# Visual-Evoked-Response-Supported Outcome of Intravitreal Erythropoietin in Management of Indirect Traumatic Optic Neuropathy

**DOI:** 10.1155/2018/2750632

**Published:** 2018-12-16

**Authors:** Mohammad Ahmad Rashad, Ahmed Abdel Meguid Abdel Latif, Hazem A. Mostafa, Samah Mahmoud Fawzy, Mahmoud Abdel Meguid Abdel Latif

**Affiliations:** ^1^Ain Shams University, Faculty of Medicine, Ophthalmology Department, Cairo, Egypt; ^2^Ain Shams University, Faculty of Medicine, Neurosurgery Department, Cairo, Egypt

## Abstract

**Purpose:**

Investigating the efficacy of intravitreal injection of erythropoietin (EPO) in managing indirect traumatic optic neuropathy (ITON) of different durations.

**Methods:**

A case series that included two groups of ITON patients: recent ITON group (<3 months trauma duration; 7 eyes) and old duration ITON group (3–36 months; 7 eyes). Diagnostic computerized tomography (CT) and baseline flash visual evoked response (VER) were performed at the presentation time. At the initial visit and each follow-up, all patients had undergone assessment of best-corrected visual acuity (BCVA), pupil reaction, and anterior and posterior segments. VER was repeated 1 and 3 months after injection. All patients received an intravitreal injection of 2000 IU EPO in 0.2 ml of commercially available sterile EPREX 4000 solution, Jansen Cilag, Zug, Switzerland. Five patients had received a second injection 3 months later.

**Results:**

Significant improvement was found in BCVA, VER amplitude, and latency (*P* < 0.0001, 0.0154, and 0.0291, respectively). Initial values of BCVA, VER amplitude, and latency correlated significantly to the final values. Differences between recent and old trauma groups were insignificant in the three parameters. In patients who received second injection, further clinical but statistically insignificant improvement was noted in BCVA in 60% of patients, VER amplitude in 50% of patients, and in VER latency in 100% of patients. No complications were recorded.

**Conclusion:**

Intravitreal injection of EPO may be effective and safe in treatment of recent and old indirect traumatic optic neuropathy.

## 1. Introduction

Traumatic optic neuropathy (TON) is a blinding form of optic neuropathies in which the incidence of no light perception (NLP) varies from 22% to 78% with variable degrees of optic atrophy [1, 2]. Direct TON can result from avulsion, transection, contusion by projectiles, or bone ships affecting the optic nerve. Indirect TON is caused by collision of the head or orbit or optic nerve against solid objects that results indirectly in compromise of blood supply by reactive vasospasm or increased intracanalicular pressure [3, 4]. Actually most of TON result from minor head trauma without orbital or skull fractures [5]. In all situations, high resolution computerized tomography (CT) is necessary to prove the diagnosis, especially in ITON by excluding compressive lesions or direct injury to the nerve by foreign bodies or bony chips [4, 6]. VER is important to confirm the diagnosis in comatosed or uncooperative patients [6–8]. Conventionally, indirect TON is managed by oral and/or intravenous high or megadose methyl prednisolone, surgical decompression of optic canal, combined therapy, or just observation. None of those lines gained universal agreement among ophthalmologists [9–13]. Since the discovery of the neurotrophic cytokine erythropoietin (EPO) human gene in 1985, [14] continuous efforts unveiled its secreting sites and its ability to maintain integrity and functions of various tissues [15, 16]. It is produced mostly from renal cells and to a lesser extent from CNS tissues. It has receptors on most CNS cell types. It was often used in hematology to promote hematopoiesis and in neurology as neuroprotective cytokine in acute lesions like stroke and traumatic brain injury to prevent apoptosis [16, 17] and in chronic neurodegenerative conditions like chronic progressive multiple sclerosis and chronic schizophrenia as a neuroregenerative agent to promote structural and functional healing [16, 17]. EPO gained access to ophthalmic field after evidence of its production in retinal cells, mainly Müller's cells [14, 18]. Reports on safety in ganglion cell protection invited experimental and human trials of using EPO in glaucomas, diabetic retinopathies, optic neuritis, and neuropathies [18–23]. Intravenous injection of EPO was also described in treatment of TON with relative success and minimal systemic side effects [24, 25]. Further studies on animal models proved safety of intravitreal injection of EPO in doses up to 5000 U by electrophysiological and histopathological examination [21, 26–29]. This promoted its use in human patients to reduce the rate of geographical atrophy enlargement secondary to age-related macular degeneration in a dose dependent manner [28] and in chronic nonresponding diabetic edema with reported subjective improvement of VA [29]. It has been also used in nonishchemic anterior optic neuropathy (NAION) with safety but limited efficacy [30].

In the few years following the Egyptian revolution in 2011, many events led to a considerable number of ocular traumas, among which direct and indirect TON ensued as conditions difficult to treat. As time passes after trauma, limitation of efficacy of conventional lines of intervention increases. Megadose steroids should be withheld if more than 8 hours has elapsed since trauma, while optic canal decompression plays no role if vision loss happened at the time of injury and not later, in addition to risk of intraoperative injury of optic nerve [1, 13]. In cases with long duration of trauma both modalities have nothing to do [1, 12]. Diversity of treatments and heterogeneity of patients' criteria encouraged us to use a relatively safer option of intravitreal injection of EPO [19, 21, 26–30] in both old and recent ITON cases. Treatment outcome will be monitored subjectively and objectively by BCVA and VER changes.

## 2. Materials and Methods

A case series included fourteen eyes of 14 patients, 13 males and one female, with ITON after either explosions or gun induced craniofacial or orbital trauma away from the optic nerve in Egypt in the period from July 2011 to July 2015.

Seven patients had trauma of less than 3 months duration, and seven were traumatized since longer time. Patients of traumas <3 months duration were still within the spontaneous improvement period [31, 32] and were considered as recent trauma group. Patients who had trauma duration ≥3 months had exceeded the spontaneous recovery period [31, 32] and thus considered as old trauma group. Demographics and clinical data of the patients are shown in Tables 1 and 2.

Inclusion criteria were as follows: history of trauma, reduced BCVA, relative afferent pupillary defect (RAPD), apparently normal or slightly pale optic disc, and delayed *P*100 latency of flash VER with reduced amplitude compared to the sound eye.

Exclusion criteria included complete or partial injury or compression of the optic nerve by bony ships or shots or hematoma as evidenced by CT, accompanying eye injuries that may account for visual reduction, follow-up shorter than 6 months, and patients with suspicious nature of TON as unclear history of trauma. We had no patient with loss of consciousness at the time of injury, blood in ethmoidal air sinuses, or received any previous treatment including steroid therapy. No exclusion was based on BCVA level or duration of trauma.

All patients and trauma witnesses were asked in detail about timing, nature, and circumstances of trauma. All cases had full ophthalmological examination including BCVA, pupil reaction, slit lamp biomicroscopy, and dilated fundus examination. They were referred to a neurosurgeon (H.A.M.) for evaluation and interpretation of high-resolution orbital and optic canal computed tomography (CT) scans performed at presentation. Baseline VER for both eyes was performed to confirm diagnosis and monitor the outcome. Hematocrit values, serum EPO level, and systemic blood pressure were measured before and after injection to monitor EPO injection safety.

Visual acuity was measured by logarithmic visual acuity Landolt's C chart in metric notation, and values less than 6/60 were measured by the technique of approximating the patient to the chart and adjusting the acuity fraction. For statistical comparisons, expression of BCVA of no light perception (NLP), light perception (LP), and hand motion (HM) was arbitrarily considered to be 3, 2.5, and 2.3 LogMAR units, respectively, similar to Kashkouli et al.'s research [24]. Increments of 0.1 LogMAR was considered a step (1 line) difference of VA [33].

After the initial clinical and radiological assessment, all patients received intravitreal injection of 2000 IU of recombinant erythropoietin alpha which is equivalent to 0.0168 mg in a volume of 0.2 ml of commercially available sterile solution (EPREX 4000, Jansen Cilag, Zug, Switzerland) in a prefilled syringe with needle guard [21, 30]. The prefilled syringe was not graded, so the desired dose was evacuated in 27 gauge graded 100 insulin syringe. Topical anesthesia was used in all patients except two of less than 16 years old who required general anesthesia. The injection was performed in operating theatre, eyelids were sterilized by povidone iodine 10% solution, and lid speculum was applied. Povidone iodine 5% solution was put on the ocular surface for 3 minutes then irrigated with saline solution. Paracentesis was made to lower IOP, and intravitreal injection of EPO was performed 3.5–4 mm posterior to the limbus. Immediate compression was applied to the site of injection to prevent EPO escape. The eye patch was kept in place till the patient arrives home. Topical ciprofloxacin and fluorometholone eye drops every 4 hours were started at the day of injection and continued for 5 days.

Postoperative assessment included BCVA measurement at the first day, 1^st^ week, 1^st^ month, 3^rd^ month, and 6^th^ month after injection. Flash VER was performed 1 and 3 months after injection. Measurements after 3 months were considered for statistics.

Five cases of improvement of BCVA ≥0.2 LogMAR units were re-injected 3 months later with the same procedure and dose. The same postinjection follow-up regimen was done although data of VER were available from 4 of the 5 re-injected cases only. All patients completed 6 months of follow-up.

The study was conducted according to the tenets of declaration of Helsinki and received the approvals of the scientific and ethical committees of Ain Shams University. A comprehensive written consent was obtained from all patients after being informed about the nature of the treatment and the possibilities of improvement.

### 2.1. Statistical Analysis

Microsoft Excel and GraphPad InStat programs were used for statistics. All data passed normality tests (parametric) and were presented as mean, standard deviation (SD), and range value. The Paired *t*-test, repeated measure ANOVA test with post hoc analysis, and Pearson's correlations were used for comparing quantitative variables. Fisher's exact test was used for qualitative assessment. Statistical significance was set at *P* value of ≤0.05.

## 3. Results

The study included 14 eyes of 14 patients with ITON, and 13 of them were males. Demographic data and trauma criteria are shown in Table 1.

### 3.1. Visual Acuity Changes

After one EPO injection, mean BCVA improved from 1.9 LogMAR (6/480 Snellen equivalent) to 1.3 LogMAR (6/120 Snellen equivalent). Total improvement ranged from 0.1 to 1.1 LogMAR (mean of 0.56 ± 0.33) that was maintained stable till the 6^th^ month follow-up. Details of BCVA changes are shown in Tables 2 and 3.

**Figure 1:**
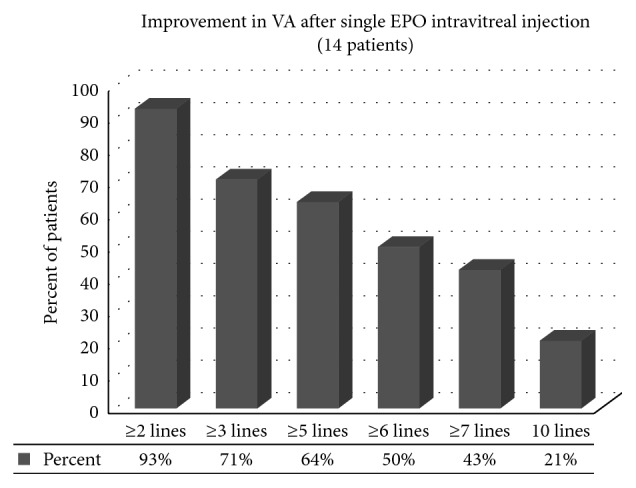
Percents of improvement in LogMAR lines after single EPO intravitreal injection (14 patients). EPO: erythropoietin.

**Figure 2:**
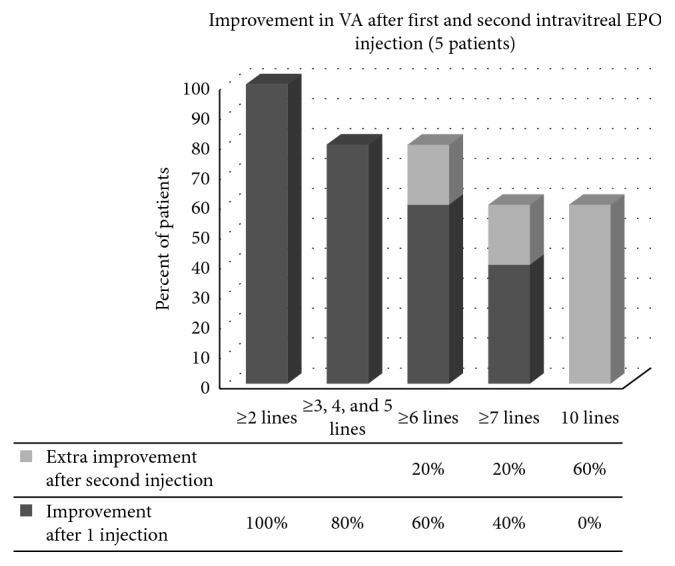
Percents of improvement in LogMAR lines after two EPO intravitreal injections (5 patients). EPO: erythropoietin.

In five of the patients who improved ≥0.2 LogMAR, a second injection was given 3 months later. After the second injection, they improved from mean of 1.5 LogMAR (6/190 Snellen equivalent) to 1.13 LogMAR (6/80 Snellen equivalent). Further gain of ≥0.3 LogMAR occurred in 3 patients (60%). Total improvement from baseline to last follow-up ranged from 0.2 to 1.5, mean of 0.93 ± 0.53 LogMAR. Comparisons between means of BCVA are shown in Table 4. Distribution of BCVA improvement in patients is shown in Figures 1 and 2.

Fifty-seven percent of patients of old trauma (4 out of 7 patients) had an improvement of ≥3 LogMAR lines after first injection, which was not statistically different from that of patients with recent traumas (6 out of 7 patients): 86%, *P*=0.56, Fisher's exact test. Three patients of old trauma (43%) gained ≥5 LogMAR lines, in comparison to 6 patients of recent trauma (86%), *P*=0.26, Fisher's exact test. Percents did not vary when considering values after second injection. Comparison of improvement of the two groups is shown in Table 5.

### 3.2. VER Changes

Baseline mean VER amplitude and latency in the ITON affected eyes were significantly worse than contralateral sound eyes (5.6 ± 6 versus 14.5 ± 15 microns, *P*=0.0018, and 157 ± 60.77 versus 117 ± 50.9 m·sec, *P*=0.009, respectively). They improved significantly 1 month after first injection and maintained the same improvement till the 6^th^ month follow-up as shown in Table 3.

VER changes in patients who received second injection and comparisons between old and recent ITON are shown in Tables 4 and 5.

**Figure 3:**
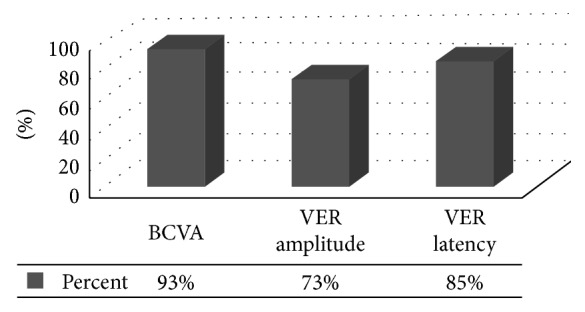
Percents of improved patients in BCVA, VER amplitude, and VER latency after EPO injection. BCVA^*∗*^: best-corrected visual acuity; VER †: visual evoked response.

Percent of improved patients in each of the three parameters is shown in Figure 3, and a decrease of 2 lines (0.2) LogMAR unit is considered as a clinical improvement.

### 3.3. Correlations between Duration, Initial Value, and Degree of Improvement of BCVA, VER Latency, and Amplitude after Injection

A weak negative nonsignificant correlation was noted between duration elapsed since injury and degree of improvement in VA (*r*=−0.349, *P*=0.24).

*Initial VER latency* was strongly positively correlated to the degree (mean) of its improvement after injection (*r*=0.86, *P*=0.0002), while *initial VER amplitude* showed no correlation to mean of its improvement (*r*=0.02, *P*=0.95). Neither of them correlated significantly to duration since injury.

In all patients, blood pressure level was stable during and after the injection, and postinjection levels of hematocrit and serum EPO showed no change from preinjection values.

## 4. Discussion

After the initial trauma, indirect nerve injury results from transmitted shearing forces to the nerve fibers or their vascular supply. Subsequent swelling of the optic nerve results in rise of intraluminal pressure and reactive vasospasm that exacerbates retinal ganglion cell degeneration [2–5]. Damage of the optic nerve can be caused by ischemia, release of harmful free radicals, bradykinins, and other inflammatory mediators followed by the cascade of retinal ganglion cells apoptosis [34, 35]. In natural history of ITON, most of the spontaneous recovery occurs during the first month, and it continues to less extent till the 12^th^ week [23, 31, 32]. According to a previous case series, spontaneous improvement occurred in 20% to 71% of cases [36, 37]. However, recovery is never complete and depends on the pathogenesis of TON [2]. Carta et al. in 2003 postulated four criteria for poor visual prognosis in ITON: loss of consciousness, blood in ethmoidal air sinuses, absence of improvement of VA after 48 hours of starting steroid therapy, and age above 40 years. In our study, we did not have the first two criteria nor used steroid treatment. And only one patient was above 40 years [38].

ITON treatments were often focused to decrease the effects of the injurious accident rather than treating the cause [39, 40]. Traditional treatment by high-dose corticosteroids harbors a risk of toxicity to the injured optic nerve and loss of life in patients with accompanying brain injury of up to 21%. The unclear benefit of optic canal decompression and the controversial outcome of steroid therapy limited the use of both in treatment of TON [9, 40].

Experimental studies on EPO proved its neuroprotective power by decreasing excitotoxicity, inflammation, oxidative stress, and harmful cell death pathways especially apoptosis [19, 29, 41, 42]. It was also found to increase survival and improve dysfunctions of retinal ganglion cells of experimental animals with induced diabetic retinopathy or glaucoma [21, 27, 29, 43]. Upregulation of EPO level for endogenous neuroprotection was found in aqueous and vitreous of eyes with glaucomatous, diabetic, and ischemic retinal conditions [18, 23, 44]. EPO mitogenic and antiapoptotic effects on the endothelium prevent ischemic retinal cell death in early diabetic retinopathy and chronic nonresistant macular edema [27, 29, 42]. However, it should be used judiciously in advanced cases to avoid aggravating proliferative diabetic retinopathy by its angiogenic property [27, 29, 42].

The neuroregenerative power of EPO was proven by promoting regrowth of transected optic nerve axons in rats through a mechanism related somehow to increasing progenitor cell proliferation [19, 45]. In the past few years, there was a great focus on its use as one of the multifunctional pharmacological therapies that activates and amplifies the endogenous restorative brain processes to promote repair, regeneration, and functional recovery [17]. It was used successfully in traumatic brain injuries and chronic neurodegenerative diseases like chronic schizophrenia and progressive multiple sclerosis [16, 41].

High doses of EPO were used to be injected systemically in neurological disorders to ensure crossing blood brain barrier [16, 17]. They were accompanied by systemic complications like increased hematocrit value, hyper active platelets, and alteration in the endothelium that predisposes patients to thrombosis [16, 17, 42]. Fortunately, in ophthalmic field, lower doses succeeded to cross blood retinal barrier without causing grave side effects [24, 25, 46–48].

Systemic intravenous administration of EPO in optic neuritis, methanol optic neuropathy, and traumatic optic neuropathy was not fully investigated, although some pilot studies reported good anatomical and functional results [24, 25, 46–48], while others did not [49].

In their studies on recent ITON, Kashkouli et al. and Entezari et al. used intravenous injection of 10.000 IU and 20.000 IU of EPO for 3 successive days and reported promising results [24, 25]. They adopted doses planned to cross blood brain barrier in CNS disorders and did not discuss feasibility to cross blood retinal barrier, nor mention the possibility of using smaller doses [24, 25, 42]. Neither of them had any serious complications [24, 25]; only 2 cases of transient hypotension during the injection were reported in Kashkouli et al.'s study [24].

Despite transient increase of EPO serum level, direct intravitreal injection of EPO in doses up to 5000 IU was proven to be safe with no increase in hematocrit value after injection [19, 21, 27, 30, 44]. Lagrèze et al. were the first to investigate the effect of intravitreal EPO in humans with occlusive vasculopathy [44]. They injected a dose of 2000 IU which is 800 times the vitreous concentration of the upregulated EPO level in retinal ischemia, which is 20 folds the level in nonischemic situations. Although they had no enough improvement in the outcome, they proved the local and systemic safety of intravitreal injection as neither blood serum EPO nor hematocrit values were elevated after the injection [44]. Modarres et al. used the same intravitreal dose in treatment of nonarteritic anterior ischemic optic neuropathy [30]. They reported improvement in visual acuity that exceeded the norm of the natural history of the disease, although it was unstable, as it declined after 3 months to settle at a level better than the initial one [30].

To the far most of our knowledge, the presenting study is the first to investigate VER-monitored efficacy of intravitreal injection of EPO in treatment of recent and old standing ITON. Unlike previous studies that reported motor vehicle accidents and falls as the major causes of ITON (>60%), [50–52] the only cause of ITON in our study was transmitted shockwaves from nearby gun shots and explosions or skull collision. Abundance of such cases following the violent events of 2011 revolution in Egypt called for definitive and accessible solutions. The apparent improvement of the recent cases in our series, in addition to recent evidences of therapeutic effect of EPO in old standing chronic neurological conditions [16, 17, 41] encouraged us to apply the same regimen to the old trauma cases. Patients of our study received intravitreal injection of 2000 IU of recombinant EPO, a dose that was proven to be safe in vitro and in vivo human studies [19, 21, 26, 27, 30, 44]. Postinjection means of BCVA, VER amplitude, and latency were significantly better than baseline values. Best-corrected visual acuity improved significantly in 93% of patients from mean of 1.9 ± 0.66 to 1.3 ± 0.85 LogMAR (*P* < 0.0001) after 1 month of injection. In the 5 patients who were injected twice, the final VA became 1.2 ± 0.83 LogMar. In all patients, no increase of hematocrit value nor serum EPO was noted, and improvement was stable till the 6^th^ month follow-up. Our results were quite encouraging when compared to research studies that used intravenous EPO in ITON like Entezari et al. who had an improvement of 72% of their patients' BCVA from 2.21 ± 0.97 to 1.48 ± 1.29 (*P*=0.001) at 1 month and to 1.31 ± 1.27 LogMAR (*P* < 0.001) at three months, also Kashkouli et al. who had an improvement in 85% of their patients' mean BCVA from 1.82 ± 1.27 to 0.94 ± 0.82 LogMAR (*P*=0.028) [24, 25]. In our study, VA improvement was supported by a concurrent significant VER improvement while neither of those research studies had a VER monitoring of their cases. Differences in patients' criteria might have accounted for the great variability in their results [24, 25], as they included wide range of initial VA with some fairly better or worse acuities than ours. The direct route of delivery of EPO in our study might also explain the relatively better outcome.

Following two injections of EPO, the NPL case in our study improved by 1 LogMAR, which was nearly similar to the three NPL patients in Kashkouli et al.'s study who improved by a mean of 0.89 ± 0.7 LogMAR, [24] but better than Entezari et al. who had an improvement of only 29% (2/7) of their NPL patients by 0.7 LogMAR [25]. Neither of those research studies had an ITON etiology other than vehicle accidents or falling, nor treated any ITON case of duration longer than 3 weeks. Kashkouli et al. reported no correlation between pretreatment time interval, which was considerably smaller than ours, and the final visual outcome of their patients [24]. In our study, we noted that it is better to correlate trauma duration to the degree of improvement in VA rather than final VA, and thus found a weak negative nonstatistically significant correlation (*r*=−0.349, *P*=0.24). We also noted a small clinical but not statistically significant difference between recent and old traumas in improvement of VA, VER amplitude, latency and in percent of patients gaining ≥3 or ≥5 LogMAR lines (*P* > 0.05).

Improvement of measurements of old duration ITON patients in our study was quite promising in comparison to the study of Acar et al. who treated 16 late stage optic neuropathy patients of mixed etiology with intravitreal EPO and concluded inefficacy of EPO treatment in improving BCVA or VER values [53].

In five patients, chosen randomly from those who gained ≥2 lines in our study, another injection of EPO was given three months after counseling the patient. Further improvement was noted in means of the three parameters in 2 patients, in VA and latency in one patient, and in VER latency only in the remaining 2 patients.

In the study carried out by Holmes and Sires on 11 acute TON patients, they postulated a cutoff VER amplitude ratio of 0.5 between the traumatized and the sound eye to be an indicator for short-term visual prognosis in natural course of the disease [8]. They stated that in patients with amplitude ratio less than 0.5, the visual acuity in the eye with TON did not exceed 20/300, i.e., about 1.2 LogMAR [8]. Their findings can be applied only to the preinjection measurements of our patients as those who had baseline visual acuity of 1.3 LogMAR or worse had an initial mean amplitude ratio of 0.45 or less, while patients who gained ≥5 lines postinjection had a worse initial ratio than those who did not (0.4 versus 0.5, respectively). Thus in medically treated cases, some factors other than initial VER amplitude might be more detrimental for possible recovery.

Amazingly, in our study, initial values of VA and VER correlated positively and significantly to their corresponding final results, although they did not correlate to the means of their improvement except for initial VER latency (*r*=0.86, *P*=0.0002). It seems that preinjection measurements can be reliable prognostic indicators for treatment outcome, especially VER latency, yet further investigations on larger scales are necessary to establish a definite biomarker for clinical improvement.

An overlook at research studies investigating TON revealed that treatment by EPO outweighs the other approaches in many aspects. Actually, fair comparisons are very difficult due to the infinite heterogeneity of patients, traumas, time of intervention, and treatment regimens. What even worsens the comparison is recruitment bias where patients with better initial VA are often dealt with by observation, while those with poor initial VA are directed towards surgery [54].

ITON observation school gives great respect to the natural history of the disease, where spontaneous improvement occurs within the first three months [31, 32]. However, visual recovery in observed cases of recent ITON is very controversial [11, 38]. Some studies reported an improvement of >0 from baseline VA in 40 to 77% of the patients [11, 38, 55, 56]. Others reported gaining ≥3 lines in about 53%, which is comparable to improvement by steroids, surgery, or both [9, 10, 57]. On the contrary, some studies found no utter improvement by observation alone [38]. More improvement was found in the present study as 86% of old and 100% of recent trauma patients had visual recovery of ≥2 lines, while 57% of old and 86% of recent traumas had ≥3 lines of VA gain. The better recovery of VA in our recent cases points to a potentiating effect of EPO to the natural healing process of early ITON course, while improvement of our old cases can be only attributed to the beneficial neuroregenerative effect of EPO as they were by far exceeding the period of spontaneous recovery. The hopeful outcome of EPO treatment may contradict other studies that reported no advantage of treatment over observation regimen alone [9, 10, 54, 57].

On the other hand, visual acuity improvement after treatment with steroids used to be controversial, unpredictable, and risky. Some researchers even deny any benefit of steroid treatment over observation [10, 11, 52, 58, 59]. By far, reported improvement with steroids is less than that found by treatment with EPO as in our study and others [10, 24, 25]. In comparison to VA gain of ≥2 lines in 93% of our study patients, a wide variation of poststeroid VA changes exists, from no improvement with IV steroids [11, 56] to an improvement of 24% to 52% of patients with oral or combined regimens [6, 9, 50, 51]. The maximum improvement reported with high-dose IV steroids in recent ITON (68.8% gained ≥4 lines) was considerably less than similar improvement in our research (85.7%) [10]. In addition to the known drastic side effects of systemic steroids, they were reported to exacerbate axonal degeneration after crash injury in rats [60]. This contradicts their presumed role in treatment of TON as compared to the proven effect of EPO in promoting axonal regeneration [19]. These controversies add up to the very limited legibility criteria of treatment with steroids, thus halting its use as a preferable line of TON treatment [6,59–62].

Unlike corticosteroids and EPO, optic canal decompression serves to remove the primary injurious agent that causes secondary axonal loss rather than decreasing the necrosis of the primary contusion or interfering with the ongoing pathological process [13, 54]. Clear evidence of fracture of optic canal, intraneural edema, or sheath hematoma should be present to ensure having benefit of surgery, even though the results are very variable [54].

VA improvement following decompression surgery alone occurs in 32% to 40% of patients [9, 13]. Delayed intervention decreases improvement to 20% [36]. While earlier intervention (within 2–5 days of injury) combined with steroid therapy augments the results to 60%–78% [55, 63]. However, early intervention was accused by confounding the results with spontaneous improvement [63]. Some researchers opted to use surgery later after failure of observation and medical treatment [32, 39]. In the presence of safer and more potent alternatives like intravitreal EPO injection, risky surgeries should be avoided to prevent serious complications like ophthalmic or carotid artery injury, CSF leak, and infection [54]. Even the results of safer endoscopic decompression were quite unsatisfactory (46% ≥1 line VA gain) [64].

The International Optic Nerve Trauma Study tried, with limited success, to unify the recruitment criteria for TON in order to have fairer comparisons. It ended up with the conclusion that neither steroids nor surgery offers the ideal solution for TON and patient-tailored treatment is advisable [9].

### 4.1. Limitations of the Study

In the current study, we had a number of limitations. Rare nature of the disease (ITON) and the difficult circumstances in Egypt at the recruitment time dictated a case series study design, where results remain uncertain due to absence of a control group. The small number of patients in each group is another limitation as we had to exclude the direct trauma cases, who were the majority of cases at that time, and cases with other ocular pathology like hemorrhage or retinal detachment. Poor control on trauma duration limited our ability to refine the classification.

Despite these limitations, the presenting study highlighted a VER-supported advantage of intravitreal EPO as a quick and safe way of delivering beneficial effects to the injured nerve. Moreover, the preliminary improvement data of VA and VER of old traumas and repeated injections encourages future research studies with control group, larger number of patients, and longer follow-up period to investigate the full therapeutic effect of EPO in direct and indirect traumatic optic neuropathies.

## Figures and Tables

**Table 1:** Patients' age and trauma duration.

		Range	Mean ± SD
Age (years)		7–50	23 ± 10.37
Duration ^*∗*^*P*=0.01	Recent trauma <3 months (7 patients)	0.5–2.7	1.24 ± 0.7
	Old trauma ≥3 months (7 patients)	3–36	16.2 ± 14.4

^*∗*^Unpaired *t*-test.

**Table 2:** Demographic and clinical data of the patients.

Age	Duration (months)	Trauma type	BCVA in Snellen's metric notation with equivalent (LogMAR)			VER: amplitude (microns)			VER latency (microns)		
			Baseline (no. 14)	After 1 injection (no. 14)	After 2 injections (no. 5)	Baseline (no. 13)	After 1 injection (no. 11)	After 2 injections (no. 4)	Baseline (no. 13)	After 1 injection (no. 13)	After 2 injections (no. 4)
31	0.5	B	NPL (3)	PL (2.5)	6/190 (1.5)	2.7	8.6	13	161	136	130.2
20	3	H.T	PL (2.5)	HM (2.3)	—	0	3.7	—	280	202	—
23	2.7	B	6/38 (0.8)	6/6.75 (0.05)	—	12.1	7.5	—	84	101	—
15	14	FB.O	6/600 (2)	6/120 (1.3)	6/60 (1)	9.3	16	17	173	169	118
7	36	B	PL (2.5)	HM (2.3)	HM (2.3)	22	32	31	150	134	127
20	36	FB.O	6/120 (1.3)	6/30 (0.7)	6/30 (0.7)	2	12.4	4.5	142	130	88
29	1.5	FB.O	6/120 (1.3)	6/12 (0.3)	—	0	3.5	—	280	111	—
18	16	H.T	6/380 (1.8)	6/300 (1.7)	—	8	6	—	160	110	—
30	1.5	FB.O	6/120 (1.3)	6/12 (0.3)	—	4	10	—	130	105	—
16	6	FB.O	6/120 (1.3)	6/60 (1)	—	—	—	—	124	102	—
50	1	H.T	PL (2.5)	HM (2.3)	—	1.6	1.7	—	107	108	—
25	1	B	PL (2.5)	6/600 (2)	—	3.55	9.17	—	95	85.5	—
13	3	B	6/190 (1.5)	6/38 (0.8)	6/8.7 (0.16)	—	—	—	155	125	—
25	0.3	H.T	HM (2.3)	6/120 (1.3)	—	—	—	—	—	—	—

BCVA LogMAR: best-corrected visual acuity in logarithm minimal angle of resolution; no.: number of patients; VER: visual evoked response. B: bullet injury to the orbit away from optic nerve. HT: head trauma from explosions or skull collision. FB.O: foreign body to the orbit away from optic nerve in bombing accident, e.g, missile particle.

**Table 3:** Clinical data of patients before and after one injection.

	Before injection (baseline)	After one injection	Paired *t*-test (*P* value)	Correlation (*r* and *P* values)
BCVA (LogMAR)			<0.0001	*r*=0.93, *P* < 0.0001
Mean	1.9 ± 0.66	1.3 ± 0.85		
Range	3–0.8	2.5–0.05		

VER amplitude			0.0154	*r*=0.829, *P*=0.0016
Mean	5.9 ± 6	10.1 ± 8.4		
Range	0–22	1.7–32		

VER latency			0.0291	*r*=0.639, *P*=0.0186
Mean	156 ± 60.77	124.5 ± 31.44		
Range	84–280	85–202		

BCVA LogMAR: best-corrected visual acuity in logarithm minimal angle of resolution; VER: visual evoked response.

**Table 4:** Comparisons of means of BCVA, VER latency, and amplitude in patients receiving two EPO injections.

	(BCVA LogMAR) 5 patients	VER latency (m·sec) 4 patients	VER amplitude (microns), 4 patients
Preinjection (baseline)	2.1 ± 0.7	156.5 ± 13.48	9 ± 9.3
After first injection	1.5 ± 0.84	142.25 ± 18	17.3 ± 10.3
After second injection	1.13 ± 0.8	115.8 ± 19.24	16.38 ± 11
Repeated measure ANOVA test	*P*=0.0033	*P*=0.0089	*P*=0.0093
Percent of patients improved after second injection	60%	100%	50%
^*∗*^Before and after first injection	*P*=0.004	*P*=0.047	*P*=0.0054
^*∗*^Before and after second injection	*P*=0.0177	*P*=0.015	*P*=0.03
^*∗*^After first and second injection	*P*=0.1156	*P*=0.11	*P*=0.77

^*∗*^Paired *t*-test *P* value. BCVA LogMAR: best-corrected visual acuity in logarithm minimal angle of resolution; VER: visual evoked response.

**Table 5:** Comparisons of improvement of BCVA, VER latency, and amplitude between patients of recent and old TON trauma.

Improvement in:	Trauma <3 months	Trauma ≥3 months	*P* value
BCVA (logMAR) (mean ± SD)	0.72 ± 0.3	0.4 ± 0.26	0.07^*∗∗*^
≥2 lines	100%	86%	0.95^††^
≥3 lines	86%	57%	0.56^††^
VER amplitude (mean ± SD)	2.76 ± 4.3	4.36 ± 4.45	0.56^*∗∗*^
VER latency (mean ± SD)	35 ± 67.5	30.3 ± 25.7	0.87^*∗∗*^

BCVA (Log MAR): best-corrected visual acuity in LogMAR. VER: visual evoked response. ^*∗∗*^Unpaired two tail *t*-test. ^††^Fisher's exact test.

## Data Availability

The data used to support the findings of this study are available from the corresponding author upon request.
